# A Method for the Assessment of Textile Pilling Tendency Using Optical Coherence Tomography

**DOI:** 10.3390/s20133687

**Published:** 2020-07-01

**Authors:** Joanna Sekulska-Nalewajko, Jarosław Gocławski, Ewa Korzeniewska

**Affiliations:** 1 Institute of Applied Computer Science, Lodz University of Technology, 90-924 Lodz, Poland; jgoclaw@kis.p.lodz.pl; 2 Institute of Electrical Engineering Systems, Lodz University of Technology, 90-924 Lodz, Poland; ewakorz@matel.p.lodz.pl

**Keywords:** optical coherent tomography, textile surface, computer image analysis, pilling grade, pilling assessment, Haralick features, texture, principal component analysis

## Abstract

Pilling is caused by friction pulling and fuzzing the fibers of a material. Pilling is normally evaluated by visually counting the pills on a flat fabric surface. Here, we propose an objective method of pilling assessment, based on the textural characteristics of the fabric shown in optical coherence tomography (OCT) images. The pilling layer is first identified above the fabric surface. The percentage of protruding fiber pixels and Haralick’s textural features are then used as pilling descriptors. Principal component analysis (PCA) is employed to select strongly correlated features and then reduce the feature space dimensionality. The first principal component is used to quantify the intensity of fabric pilling. The results of experimental studies confirm that this method can determine the intensity of pilling. Unlike traditional methods of pilling assessment, it can also detect pilling in its early stages. The approach could help to prevent overestimation of the degree of pilling, thereby avoiding unnecessary procedures, such as mechanical removal of entangled fibers. However, the research covered a narrow group of fabrics and wider conclusions about the usefulness and limitations of this method can be drawn after examining fabrics of different thickness and chemical composition of fibers.

## 1. Introduction

Pilling arises as a result of mechanical factors during the day-to-day use of fabrics, mainly friction that occurs when the fabric rubs against another surface. Resistance to pilling is desirable from the points of view of both fabric durability and aesthetics. The main characteristic of pilling is the formation of small knots at the ends of protruding fibers, which negatively affect the appearance of fabrics. Pilling is generally considered to be a self-limiting process that occurs in several stages. Studies by Gintis and Mead [1] have demonstrated that pilling involves three distinct phases, the first two of which are connected with pill formation. First, the fibers are drawn to the fabric surface as a result of mechanical action, resulting in the formation of fuzz. The fuzz then becomes entangled into pills. The final stage is the loss of pills under continued mechanical action, such as rubbing, laundering, or drying. Cooke [2] supplements the description of the pilling process with more detailed intermediate steps. After the establishment of a localized area of high fuzz density, he distinguishes a phase of developing entanglement within that area. The tangle is then compressed into a roughly spherical mass of fiber, anchored to the fabric by a few unbroken fibers. When the pills form, they are connected to the fabric. However, the anchoring fibers are subsequently pulled out due to abrasive forces. As a result, discrete mobile pills are formed. During further use of the fabric, some of the anchors may become fractured. This process proceeds until all the pills are shed.

In practice, the level of pilling should be specified according to the rates of the parallel processes, i.e., fiber entanglement leading to pill formation, the development of more surface fiber, and fiber and pill wear-off. The rates of these processes depend on the properties of the fiber, yarn, and fabric [1,3]. Extreme cases concern fabrics containing strong fibers and fabric containing weak fibers. In the case of fabrics with strong fibers, the rate of pill formation exceeds the rate of wear-off. This results in increased pilling, and an increase of wear. In the case of fabrics with weak fibers, the rate of pill formation competes with the rate of wear-off. This may result in variable pilling symptoms, and an increase of abrasion. There are other constructions in which surface fiber wear-off occurs before pill formation. Each of these examples demonstrates the complexity of evaluating surface changes on different types of fabric. Moreover, due to the action of the pilling motion, fabric surfaces contain pills of different size and fuzziness. Hence, to better understand the pilling process fabric surface variations need to be evaluated as a whole.

The assessment of pilling resistance carried out in textile laboratories relies on intensive, standardized abrasion of specimen surfaces with a brush, followed by a subjective quantitative assessment of the amount of abrasion or pilling, according to a five-grade rating scale (ASTM D 3511-08). Grade 5 corresponds to pilling-resistant material, while grade 1 indicates very severe pilling. Grade 3 indicates moderate surface pilling. According to PN-EN ISO 12945-2:2002, grade 3 is an acceptable rating for both woven fabrics and knitted fabrics after 2000 rubs. The same rating is acceptable according to PN-EN ISO 12945-1:2002 after 7200/14,400 revolutions. According to these standards, the tested samples should also be assessed in terms of the distribution of pills on the surface and the occurrence of other changes affecting the aesthetic values of the material (e.g., color change). Grading is usually performed in pilling assessment chambers, under an appropriate fluorescent light source. The sample is compared to a standard photograph or a reference sample to estimate the number of pills.

The disadvantages of this method include the approximate nature of the results and the risk of incorrect measurement due to the nature of the fabric. Pilling tests using standard abrasion methods do not take into account artifacts in the construction of the fabric, nor falsification of the results by pills detached due to prolonged rubbing.

To improve the quality and durability of textiles, many studies have attempted to identify pilling patterns automatically, by means of image processing technology. Some non-intrusive methods use 3D techniques, such as a laser profilometer [4] or high-resolution camera scanning system [5]. These techniques provide data on the roughness of the scanned surface, which may also be assessed in terms of pilling intensity. A three-dimensional fabric image obtained with a laser beam can also be analyzed to determine the number, area, and distribution of pills [4,6]. Recently, pilling assessment methods using thresholding algorithms [7,8], Fourier’s analysis [9,10], and artificial intelligence [11,12] have become more popular both for two- and three-dimensional images.

This paper describes a new approach to assessing the pilling tendency of textiles, based on short-term abrasion tests. These abrasion tests do not tend to cause pills, but only lead to the early stage of fiber detachment. Using 3D images of the near-surface area of the textile after the test, the fibers protruding from knitwear are observed and analyzed. To detect the pixels of fiber objects, we apply the methods of image analysis to evaluate quantitative and textural indicators of pilling intensity. The surface of the knitwear is mapped by means of infrared light, which is emitted, penetrates the material, and is recorded after reflection from the surface based on the principles of optical coherence tomography (OCT) [13]. This technology is a powerful tool, which enables sensitive inspection of surfaces and fiber arrangements with ≈5μm resolution. OCT is one of the most innovative and rapidly emerging optical imaging techniques in the last decades. Potential fields for OCT applications are steadily increasing through both numerous functional and contrast-enhanced [14] extensions of OCT and combining OCT into multi-modal optical systems [15]. For example, the combination of OCT, Raman spectroscopy and optoacoustic provides both morphological and molecular information from a target biological tissue [16]. All three modalities combined have the potential to provide a kind of non-invasive optical biopsy for skin cancer screenings in deeper lesions. OCT has recorded the most applications so far in medicine, especially in ophthalmology. Although ophthalmology is currently almost mainstream of OCT application, this technique is still being developed in this field [17].

Chu et al. developed the ultrahigh-speed SS-OCT handheld system with a 2D micro-electro- mechanical system (MEMS) scanning mirror for ophthalmic retinal imaging [18]. Applications of 2D MEMS and OCT in real-time medical imaging were also investigated by Cogliati et al. [19].

Already highly established in medical applications, this non-destructive and contactless technique is being extensively investigated, at the moment, in material sciences for determining the microscopic properties, such as thickness, roughness, and surface profile of steel materials [20,21,22], laser-processed plastics [23] and composites [24,25]. Recently, OCT was implemented for recognition of weave patterns of fabrics [26,27].

Analysis of texture content in digital images plays an important role in the automated visual inspection of textile images. Such approaches are used mainly to assess the properties and quality of fabrics, including flaw detection [28] and surface structure [29]. The most popular method for quantitative assessment of texture is the use of Haralick features [30,31]. These features are calculated from a Gray Level Co-occurrence Matrix (GLCM), which counts the co-occurrence of neighboring gray levels in the image. Haralick texture features were originally defined for 2D images, but in this study we use spatial images of the pilling layer over the fabric surface, formed by pills and single, protruding fibers.

## 2. Materials and Methods

### 2.1. Fabric Material and Laser Processing

Three types of left-right weave knitted fabrics were tested to determine their pilling propensity. The first sample (F1) was a polyester textile of the single jersey type. The second fabric (F2) was a polyester-polyacrylonitrile knitwear known as Lacosta blue. The third textile was cotton-poliamid knitwear (F3). All the fabrics had a surface density of 240 $g/m^{2}$.

The analyzed fabrics were characterized by high susceptibility to pilling. This tendency was observable as large amounts of entangled fibers clinging to the cloth surface, which appeared when the fabrics were worn as clothing. However, despite their common high pilling tendency, the fabrics were rated differently according to the ISO-12945-2-200 standard (Table 1).

Some of the samples were subjected to laser modification, to reduce their tendency for pilling. Laser modification leads to the deposition of nanoparticles and nanocrystallinity, which bonds the fibers on the fabric surface [4]. The surfaces of the samples were modified by laser ablation at different laser powers, taking into account features of the fabric such as structure and color (Table 1). A 20 W single-mode optical fiber laser was used with a wavelength of 1.064μm, manufactured by SPI (Southampton Photonics Inc.) Lasers company. Unmodified and modified knitwear was subjected to the same set of abrasion tests. The purpose was to determine the suitability of the proposed pilling indicators for various fabric cases. A laser pulse with a frequency 80kHz was applied with a duration of 55ns. The laser beam scanned the surface of the textile material at a speed of 400mm/s with hatching 0.01mm [32].

### 2.2. Abrasion Tests

**Figure 1:**
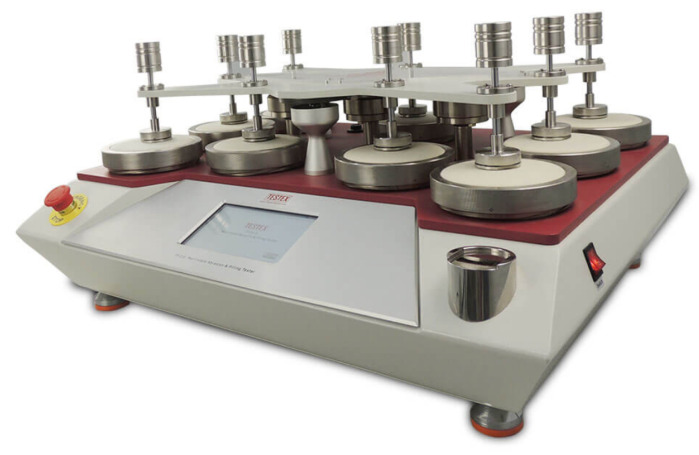
Martindale Abrasion and Pilling Tester TF210 [33].

We used a Martindale device (Figure 1) [33] and the principles of forced pilling described in PN-EN ISO 12945-2:2002 and PN-EN 12947-1:2002. Samples with a diameter of 14cm were mounted on a felt disk on the head of the device. A felt disc with the tested material placed on it was also mounted on an instrument table. The disc was moving at a constant speed. Tests were carried out under a load of 415g (typical for fabric testing). The head moved relative to the stationary table along a Lissajous curve track. The samples were evaluated after 5000 head movements. Due to the subjective nature of the assessment, the samples were evaluated independently by three people according to ASTM D 3511-08. The presented results are the arithmetic averages of the partial grades given by the evaluators.

To cause fiber protrusion, which precedes other typical symptoms of pilling in knitted and woven fabrics, each type of fabric material was manually rubbed using a rough surface for 15s while maintaining a constant pressure. Two types of surfaces were used for fabric friction testing—a brush with hard fibers (Test 1) and an unglazed ceramic plate (Test 2). The pressure force of the friction surface on the material, controlled by a strain gauge, was set at 2N Test 1 and 1N for Test 2.

Immediately after the abrasion tests, the surface of the fabric was scanned in places subjected to abrasion using OCT, and the three-dimensional OCT images were archived for further numerical analysis of the pilling symptoms. After each test, five volumetric OCT images were made for each of 3 types of fabric without (F1, F2, F3) and for 3 types of fabric after laser treatment ($F1^{a}$, $F2^{a}$, $F3^{a}$). Every fabric case (F1, F2, F3, $F1^{a}$, $F2^{a}$, $F3^{a}$) was considered to be the untreated fabric (control one) and after 3 abrasion tests. The data set contained a total of 120 OCT images of the control and abrasion-tested fabrics.

### 2.3. Textile Image Acquisition and Pre-Processing

**Figure 2:**
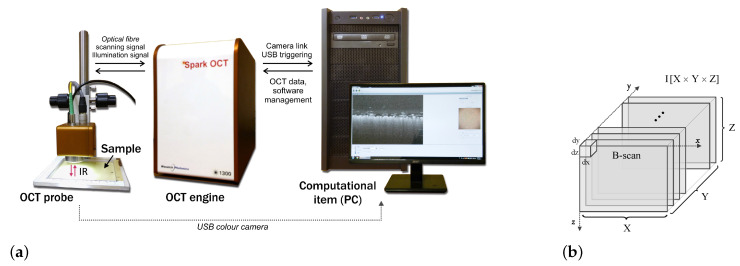
Acquiring images of textile fabric (**a**) The functional modules of Spark OCT 1300nm system; (**b**) the stack of acquired B-scans equivalent to volumetric data.

A Spark OCT-1300 image acquisition system by Wasatch Photonics Inc. [34] was used to register infrared spatial images of the measured fabric layer. A block diagram of the system is shown in Figure 2. The device consists of three main modules: an OCT Engine, an OCT Imaging Probe, and a Computational Engine. The OCT Engine contains a Michelson interferometer, electronics, a spectrometer, a reference arm, and polarization and path length controllers. The interferometer uses a low coherence broadband laser source and a spectrometer transmitting an image from diffraction grating typically for spectral domain OCT (SD-OCT). The engine emits an infrared laser beam centered at a wavelength of 1300nm. The scanning depth can be calculated directly from the acquired Fourier transform spectra, without movement of the reference arm. The OCT Imaging Probe contains a light source, scanning mirror, optics, and a color camera for creating en-face images of the scanned region. The computational engine is a PC including a Camera Link card for acquiring data from the spectrometer and receiving synchronization triggers from the engine. Once the infrared laser light has penetrated the region of the textile fabric, the intensity of the signal reflected in the location (x,y,z) is stored in the form of a three-dimensional image array in the PC memory.

Each image (volumetric data) covers a maximum volume 0.5×0.5×0.4cm containing both the fabric layer and the pilling layer above it. This space corresponds to a raster of 512×512×640 voxels in the Cartesian coordinate system OXYZ. Any image voxel illustrated in Figure 2b has the dimensions equal to dx=5.1μm, dy=4.8μm and dz=5.4μm. In the experiments carried out, to limit the amount of data processed, low resolution images were acquired, where lateral resolutions are 2dx and 2dy. The scanning process provides B-scan frames (OXZ-planes) in real time and creates a full 3D image from these frames in off-line mode. The operation takes around 20s. To simplify and accelerate the proposed algorithm, it is assumed that the scanning head is positioned so as to maintain the fabric layer in a horizontal orientation inside each B-scan.

### 2.4. Fabric and Pilling Layer Detection

**Figure 3:**
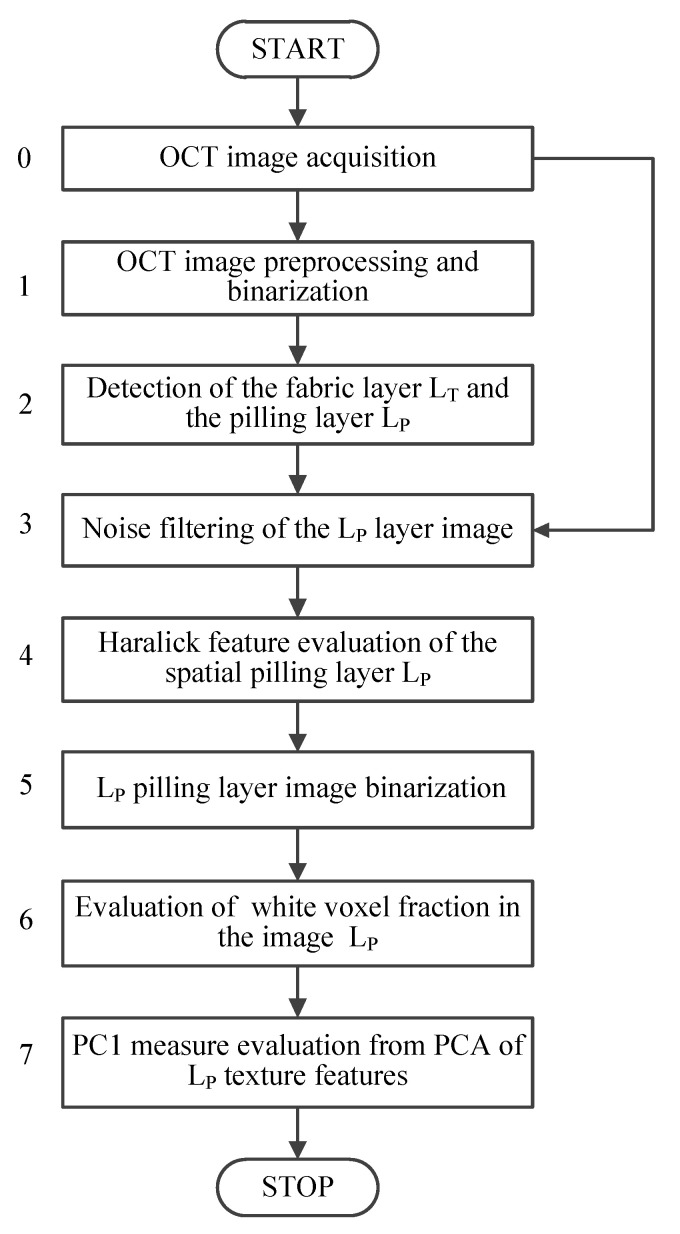
Flowchart of the proposed algorithm.

The goal of the image processing algorithm is to extract the pilling fiber layer located above the fabric surface as a separated image. The pilling image is then subjected to textural analyses, providing quantitative indicators of the degree of pilling (Figure 3). The algorithm is designed for the Python 3.6 environment working in a Windows 10 operational system [35,36].

**Figure 4:**
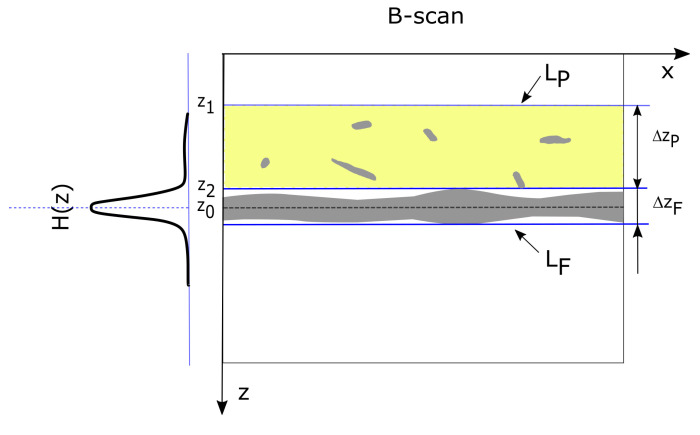
Layers inside the B-scan and their Hough transform in the horizontal direction *x* (at an angle of $90^{{}^{\circ}}$): $L_{P}$—pilling layer; $L_{F}$—fabric layer; $\Delta z_{P}$—height of $L_{F}$ layer; $\Delta z_{F}$—the height of $L_{F}$ layer; H(z)—the Hough transform of a B-scan in the horizontal direction; $z_{0}$—the middle line position of the fabric layer $L_{F}$; $z_{1},z_{2}$—limits of the pilling layer $L_{P}$.

Block 1 of the algorithm flowchart in Figure 3 shows the pre-processing step. The core fabric layer is extracted from the OCT image. Visible pilling fibers are suppressed using the concatenation of image opening and mean filtering operations. Pre-processing starts with the morphological opening presented in Equation (1) [35,37],

(1) $$I^{\prime }\left(x,y,z\right)=\left(I\circ S_{C}\right)\left(x,y,z\right),$$

where *I* is the processed OCT image, ∘ is the symbol of the opening operation, $S_{C}$ denotes a cube-shaped structuring element with an edge length of 3 pixels. It is used for morphological noise removal and eliminates small objects to facilitate detection of the fabric layer $L_{F}$. The opening is a concatenation of minimum and maximum low pass image filtering. The partial operations replace each image value I(x,y,z) by the minimum or maximum value found in the $S_{C}$ neighborhood of (x,y,z). As a result of the opening, small bright areas of the OCT image on a dark background are removed so as not to interfere with the detection of the fabric layer visible as a large horizontally located bright area. In the Python implementation, the opening was performed using the function $skimage.morphology.opening(I,S_{C})$, where $S_{C}$ = skimage.morphology.cube(3). Next, an averaging filter was applied to each B-scan, according to Equation (2):

(2) $$I^{\prime }\left(x,y,z\right)=\left(I*W_{A}\right)\left(x,y,z\right)$$

where $W_{A}$ is the local filtering window [5×5×1] pixels in X,Y,Z directions with the unit weights of each pixel. The convolution $I*W_{A}$ is executed in the Python script as $scipy.ndimage.uniform\_filter(I,W_{A})$. The OCT image filtering sequence given above exposes the core fabric layer, the location of which can be detected easily after thresholding to the binary image $I_{B}$. The proposed thresholding is performed globally with a threshold value of $T_{1}$, obtained by the Otsu method [38] described in Equation (3):

(3) $$I_{B}\left(x,y,z\right)=\left\{\begin{array}{cc} 1 & I\left(x,y,z\right)\geq T_{1},\\ 0 & \mathrm{otherwise} \end{array}\right.$$

where $I_{B}$ denotes the resulting binary image, and $T_{1}$ is evaluated by the Python function skimage.filters.threshold_otsu(I). In each B-scan of the binary image $I_{B}$, the searched fabric layer is represented by a single horizontal object with a different width or by the set of disjoint binary objects inside the horizontal band $\Delta z_{F}$, as illustrated in Figure 4.

To detect the middle line of the fabric layer $L_{F}$ in a B-scan, rare pilling fiber objects outside that layer should be omitted, while binary objects in the scope $\Delta z_{F}$ that form the layer core should be included. The Hough transform [39] fulfills this requirement, when limited to the detection of a horizontal line in every XZ-plane. In the considered case, the Hough transform can be simplified to the form given in Equation (4).

(4) $$\underset{y}{\forall }\;H\left(z\right)=\sum_{x}I_{B}\left(x,z\right)$$

The transform H(z) is smoothed by the averaging window $W_{z}$ to eliminate unwanted high frequency fluctuations over *z*. This facilitates detection of the fabric layer limit.

(5) $$H^{\prime }\left(z\right)=\left(H*W_{z}\right)\left(z\right).$$

The filter in Equation (5) is implemented as a Python function scipy.ndimage.uniform_filter() with a $W_{z}$ window 13 pixels wide. This pixel width was adjusted experimentally. Next, the fabric layer middle line position $z_{0}$ is determined as the average value H(z) according to Equation (6).

(6) $$z_{0}=\sum_{z=0}^{Z-1}zH\left(z\right)/\sum_{z=0}^{Z-1}H\left(z\right)$$

**Figure 5:**
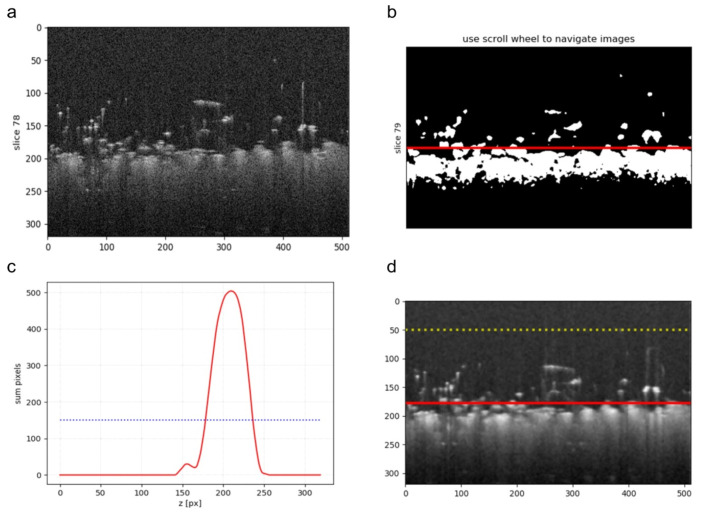
Illustration of pilling layer extraction steps for a knitwear B-scan: (**a**) example B-scan image; (**b**) the image from figure (**a**) binarized using the Otsu method with the detected knitwear surface line; (**c**) the horizontal Hough transform H(z) of the binary image in figure (**b**) with the line of the material surface detection level $k\cdot H(z_{0})$ (Equation (6)); (**d**) the image from figure (**a**) with the pilling layer detected between horizontal lines.

The upper limit of the fabric layer is determined in relation to $H(z_{0})$, as shown in Equation (7):

(7) $$z_{2}=max\left\{z:\;z<z_{0}\;\;\wedge \;\;H\left(z\right)\leq k\cdot H\left(z_{0}\right)\right\},$$

where k=0.3 is the ratio of H(z) reduction outside the fabric middle line, which was selected experimentally, $z_{2}$ is the maximum value in the set of *z* values that are less than $z_{0}$ and for which $H\left(z\right)<kH\left(z_{0}\right)$. The $z_{2}$ horizontal line represents the upper limit of the fabric layer equal to the lower limit of the searched pilling layer. In all B-scans, the pilling layer has the same height $\Delta z_{P}=z_{2}-z_{1}$, selected experimentally for all tested fabrics. Therefore $z_{1}=z_{2}-\Delta z_{P}$ is the value computed form $z_{2}$ and $\Delta z_{P}$. The set of regions $\Delta z_{P}\left(y\right)$ in each B-scan y=0,…,Y−1 defines the cuboid of the pilling layer $L_{P}$.

(8) $$L_{P}\left(x,y,z^{\prime }\right)=\left\{\left(x,y,z\right):z\in [z_{1}\left(y\right),z_{2}\left(y\right)]\right\},$$

where $z^{\prime }=z-z_{1}$. The above operations for separating layers correspond to block 2 of the algorithm in Figure 3. Figure 5 presents exemplary results of the processing stages discussed above. Figure 5b includes binarized B-scan obtained from the block 1 of the algorithm flowchart in Figure 3 with the detected knitwear surface line evaluated in the block 2, corresponding to the value $z_{2}$ in Equation (7). Figure 5c is the plot of H(z) for the binarized B-scan. Figure 5d contains the final result of the algorithm block 1 and 2—the limits $z_{1}$ and $z_{2}$ of the $L_{P}$ layer visible as dotted and continuous line respectively

The next step of the algorithm is independent of pre-processing in the blocks 1 and 2 of the algorithm shown in Figure 3. It contains median filtering [40] of the input image *I*, expressed in Equation (9):

(9) $$J\left(p\right)=\underset{q\in W_{M}\left(p\right)}{median}\left\{I\left(q\right)\right\},\;p,q\in I,$$

where $W_{M}\left(p\right)$ denotes the cuboid window of dimensions $\left[W_{X}\times W_{Y}\times W_{Z}\right]$ around each voxel p=(x,y,z) of the image *I*. This is a necessary step before pilling analysis, to reduce the speckle noise inherent in the content of OCT images. Filtering can be performed by a Python script with the function scipy.ndimage.median_filter(), applying the local window [5×5×5] voxels.

### 2.5. Assessment of Pilling Layer Textural Features

The median filtered image $I_{P}$ of the layer $L_{P}$ above the fabric surface without pilling is composed almost exclusively of dark voxels, corresponding to a homogeneous air layer of constant and low density. In the presence of pilling, fibers projecting above the fabric surface are damp and dissipate the OCT infrared radiation, resulting in many areas of bright pixels in the image $I_{P}$. The surfaces and numbers of these areas increase with the amount of migrating material, in the form of individual fibers or bundles of fibers. Therefore, a possible measure of the degree of pilling is the increase in the number of bright pixels in the image $I_{P}$ and the appearance of large changes in the brightness of adjacent pixels, which will imply changes in the image $I_{P}$ texture. The speckle noise errors are reduced by median filtering expressed in Equation (9).

The full OCT image *J* (with fabric and pilling layers), denoised by the local median in Equation (9), is binarized with a global brightness threshold in Equation (10), similarly to Equation (3):

(10) $$J_{B}\left(x,y,z\right)=\left\{\begin{array}{cc} 1 & J\left(x,y,z\right)\geq T_{2},\\ 0 & \mathrm{otherwise} \end{array}\right.$$

The computed Otsu threshold $T_{2}$ defines the fraction of pixels with brightness similar to the fabric layer. This corresponds to the outlying fibers of pilling in the $L_{P}$ layer. The fraction $f_{P}$ is evaluated as

(11) $$f_{P}=\frac{1}{V_{P}}\sum_{x,y,z}J_{B}\left(x,y,z\right),$$

where $z\in [z_{1}\left(y\right),z_{2}\left(y\right)]$ belongs to the layer $L_{P}$ and $V_{P}$ is the volume of the layer measured in voxels.

The content of the pilling layer image $L_{P}$ is characterized by Haralick textural features [30,31]. The features are computed based on the gray level spatial dependence matrix (GLCM) at a distance of *d* image voxels. The features are averaged for 13 directions in an image space determined by side and diagonal neighborhood. Five features were selected to characterize pilling in the image $I_{P}$: $H_{1}$ (Energy), $H_{2}$ (Contrast), $H_{4}$ (variance), $H_{5}$ (Homogeneity), and $H_{9}$ (Entropy).

(12) $$H_{1}=\sum_{i=1}^{N_{g}}\sum_{j=1}^{N_{g}}p{\left(i,j\right)}^{2},$$

where $N_{g}=256$ is the number of gray levels, and p(i,j) is (i,j) entry in a normalized GLCM defined for each pair of related voxels at a distance d=2. According to the Haralick original paper [30] p(i,j) means the relative frequency with which two pixels separated by a pixel distance *d* in one of the space directions occur, one with intensity *i* and the other with intensity *j*. The energy feature $H_{1}$ of the GLCM is related to the brightness repeatability of associated pairs of voxels. The high value of $H_{1}$ in a uniformly dark image with no pilling fibers will decrease as the layer $L_{P}$ fills with the bright pixels of protruding fibers.

(13) $$H_{2}=\sum_{n=0}^{N_{g}-1}n^{2}\left\{\sum_{i=1}^{N_{g}}\sum_{j=1}^{N_{g}}p\left(i,j\right)\right\},\;\left|i-j\right|=n,$$

The feature $H_{2}$ defined in Equation (13) provides a measure of contrast or local intensity variation. It prefers contributions of p(i,j) located away from the GLCM diagonal, i.e., i≠j. The $H_{2}$ contrast feature sums voxel pairs with constant differences in brightness levels, which are additionally weighted by the size of the differences. Its value increases in the presence of many white and black neighboring voxels around individual material fibers. The variance of voxel brightness co-occurrence is expressed by the $H_{4}$ feature, defined by Equation (14):

(14) $$H_{4}=\sum_{i=1}^{N_{g}}\sum_{j=1}^{N_{g}}{\left(i-\mu \right)}^{2}p\left(i,j\right),$$

where μ represents the average value of GLCM. The feature $H_{5}$ in Equation (15) provides a measure of the homogeneity in an intensity image. It returns a value that corresponds to the closeness of the distribution of elements in the GLC matrix to its diagonal.

(15) $$H_{5}=\sum_{i=1}^{N_{g}}\sum_{j=1}^{N_{g}}\frac{p(i,j)}{1+{\left(i-j\right)}^{2}}.$$

Homogeneity $H_{5}$ is equal to 1 for a diagonal GLC matrix. The $H_{4}$ feature value increases and $H_{5}$ decreases with the variety of gray levels above the fabric surface accompanying the phenomenon of pilling. Another Haralick feature known as the sum average $H_{6}$, used later in the article as an auxiliary property is described by the following relation:

(16) $$H_{6}=\sum_{k=2}^{2N_{g}}k\;p_{x+y}\left(k\right),\;p_{x+y}\left(k\right)=\sum_{i=1}^{N_{g}}\sum_{j=1}^{N_{g}}p\left(i,j\right),\;i+j=k.$$

In Equation (16) $p_{x+y}\left(k\right)$ denotes the cumulative frequency of image pixel pairs with constant sum of intensities equal to *k*. $H_{6}$ is the average value of these cumulants for all possible intensity sums $k=2,3,\ldots ,2N_{g}$. Image entropy $H_{9}$ presented in Equation (17) takes small values for homogeneous scenes and greater values when the heterogeneity of the image brightness increases.

(17) $$H_{9}=-\sum_{i=1}^{N_{g}}\sum_{j=1}^{N_{g}}p\left(i,j\right)\times log\left(p(i,j)\right),$$

In the proposed algorithm, the function $mahotas.features.haralick(J_{P},distance=2)$ computes the Haralick texture features in the three-dimensional image $J_{P}$ of the pilling layer $L_{P}$. Because the values of some features may appear on a very different scale, they have been normalized for better comparison. The normalization transforms the feature components into random variables with zero mean and unit variance, using the formula given in Equation (18) [41]:

(18) $${\tilde{H}}_{x}=\frac{H_{x}-\mu \left(H_{x}\right)}{\sigma (H_{x})},$$

where $\mu (H_{x})$ and $\sigma (H_{x})$ are the sample mean and the sample standard deviation of the feature $H_{x}$. The $H_{x}$ sample set, for which the statistics μ and σ are computed, includes values either before or after any type of abrasion test is carried out. This emphasizes the feature changes resulting from fabric treatment.

Principal component analysis (PCA) was applied to all textural data obtained from the samples, to reduce the high dimensionality of feature space while preserving the quantitative differentiation of the results for different levels of pilling [42].

## 3. Results

### 3.1. Pilling Visual Assessment

**Figure 6:**
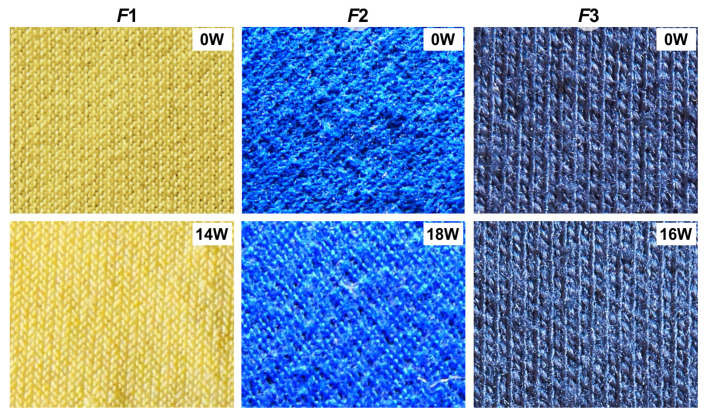
Knitwear surfaces after the standardized Martindale pilling test: 0 W indicate fabrics without laser treatment; 14–18 W indicate fabrics after laser ablation.

Figure 6 provides a visual comparison of the textile samples subjected to abrasion using a Martindale instrument. The tests revealed varying degrees of textile pilling, depending on the types of fabric and surface pretreatment. As can be seen, the flock fibers of sample F1 were almost completely removed from the substrate fabric during the test cycle. Based on visual inspection, specialists assigned this fabric pilling grade 5. In the case of the other two example fabrics (F2 and F3), standardized friction tests resulted in the entanglement of fibers into balls over the fabric, along with residual free fibers. The cardinality of these phenomena corresponded to grade 2 and grade 2–3 of pilling, for fabrics F2 and F3 respectively. Modification of the fabric surface by laser ablation revealed fuzzing due to abrasion in fabric F1 and it caused a slight reduction in pill number in fabrics F2 and F3. Information on the pilling grades of these fabrics, including after laser ablation, is given in Table 1 (Section 2.1).

**Figure 7:**
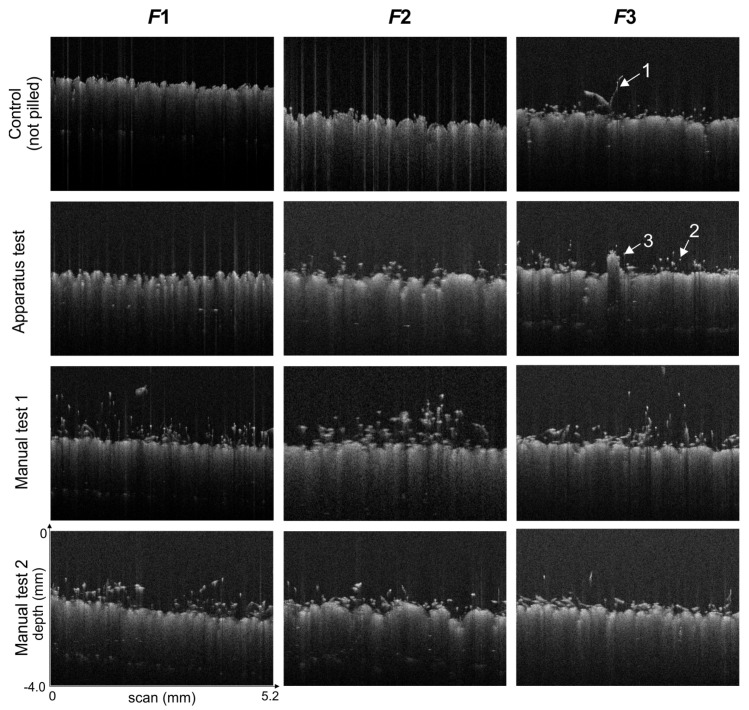
Exemplary two-dimensional OCT images (B-scans) of knitwear cross-sections taken from volumetric data, illustrating the appearance of a layer above the fabric in the absence of pilling (control samples of F1 and F2, and F1 after standardized abrasion test), in the early stages of pilling (all tested fabrics after manual tests), and during the pilling phase after standardized abrasion test (F2 and F3). 1—loose fabric fibers, 2—cut fiber fragments, 3—a pill attached to the fabric surface.

Figure 7 shows example OCT images corresponding to cross-sections through the fabrics after the Martindale test and manual tests. The pilling symptoms in the OCT images usually appear in the form of white pixels, belonging to protruding fibers and pills.

As can be seen, the standard long-term pilling test did not result in pilling symptoms in the case of the knitwear sample F1, but manual short-term tests resulted in fiber detachment, i.e., the initial pilling phase, including an area of high fuzz density. In the cases of the other two fabrics (F2 and F3), the number of fiber pixels increased compared to the original fabric after both manual and machine abrasion tests. In the OCT images of these fabrics, after the Martindale test both pixels in formations (pills) and loose pixels forming fiber fuzz can be observed. Manual tests pulled the fibers above the fabric plane. Test 1, using a rough friction surface, caused both fuzz formation and the development of entanglement, while the less invasive Test 2 led only to the formation of fuzz.

### 3.2. Pilling Feature Analysis in OCT Images

Table 2 and Table 3 include the values of the voxel fraction $f_{P}$ and selected Haralick features for the pilling layer $L_{P}$ obtained as a result in step 4 and step 6 of the algorithm illustrated in Figure 3. In each abrasion test conducted in the experiment a fabric sample was imaged five times in various places under the same condition. Tables display the mean values of pilling measurements of the sample in the repeated tests.

In Table 3 only $H_{1}$, $H_{2}$, $H_{4}$, $H_{5}$, $H_{6}$, $H_{9}$ textural features were cited from the set of all 13 calculated Haralick features. These features were chosen by the authors because their changes after abrasion tests can be predicted looking at the OCT images of the pilling layer as in Figure 7, or changes in their value have no significant relationship to the density of fibers in the pilling layer ($H_{6}$). According to the meaning of those features described by Equations (12)–(17), the contrast ($H_{2}$), variance ($H_{4}$) and entropy ($H_{9}$) should grow with the number of regions including neighboring bright and dark pixels. This takes place when many fibers pulled from the fabric layer by abrasion tests migrate to the $L_{P}$ layer. On the contrary, energy ($H_{9}$) or homogeneity ($H_{5}$) should then decrease because the $L_{P}$ layer brightness becomes uneven as can be seen in Figure 7.

**Figure 8:**
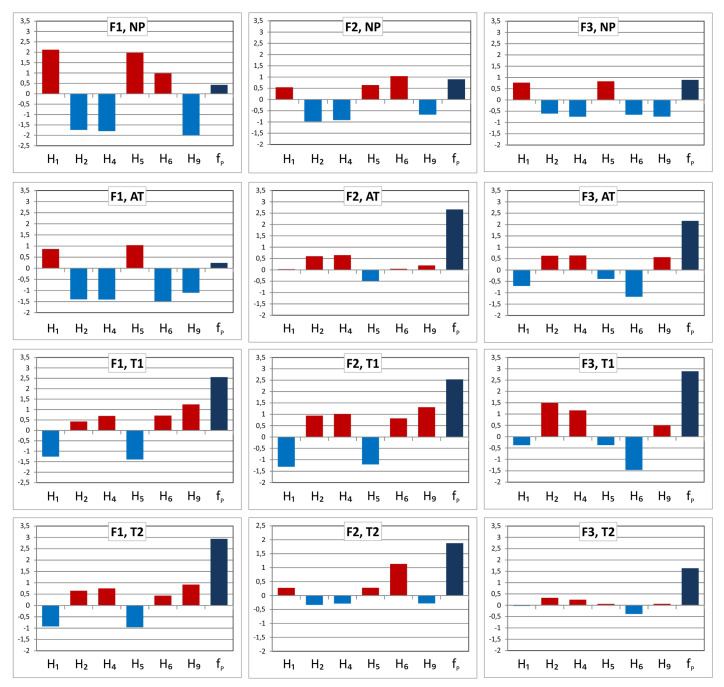
Selected texture feature variability due to fabric abrasion: F1, F2, F3—types of fabric tested; $H_{1}\;\left(energy\right)$, $H_{2}\;\left(contrast\right)$, $H_{4}\;\left(variance\right)$, $H_{5}\;\left(homogeneity\right)$, $H_{6}\;\left(sum\;average\right)$, $H_{9}\;\left(entropy\right)$—Haralick feature types; $f_{P}$—fraction of fiber pixels; NP—not pilled (control) sample; T1, T2—manual tests; AT—apparatus test.

The texture Haralick feature values were normalized according to Equation (18) to obtain comparable ranges for various features and prepare them for further processing. Figure 8 visualizes the changes of the selected features under different tests for a given fabric type (F1, F2 or F3). Considering a specific fabric type it is easy to see that the features $H_{2}$, $H_{4}$ and $H_{9}$ change similarly under the influence of all proposed tests (T1, T2, AT). The same can be said about the behavior of $H_{1}$ and $H_{5}$ although they change in the opposite direction than the first group of features. Only the feature $H_{6}$ varies without any relation to the other. This suggests that all selected features except $H_{6}$ are correlated and can be approximately represented by one common pilling indicator.

To reduce space dimensionality of the measured parameters and to verify correlation degree among original features PCA transform is applied. This operation reveals the internal structure of the data and best explains the variance in the data. It maps the features to a new coordinate system such that the greatest data variance lies on the first coordinate called the first principal component PC1, the second greatest variance on the second coordinate PC2, and so on.

**Figure 9:**
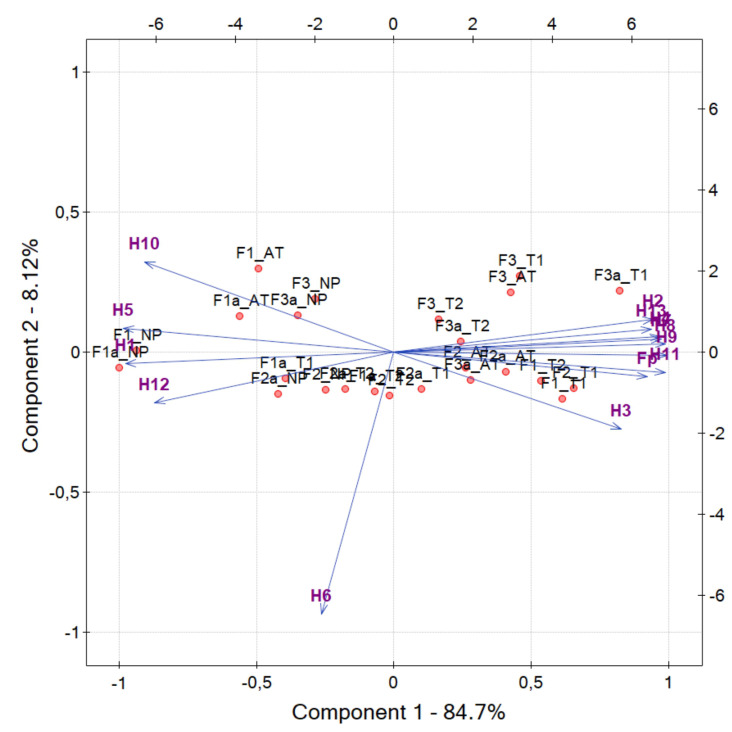
Score plot for *PC*1 and *PC*2 for different fabric abrasion tests, where: F1, F2, F3—tested fabrics; NP—not pilled (control) sample; T1, T2—manual tests; AT—apparatus test. A complete set of Haralick’s texture features ($H_{1}$–$H_{13}$) calculated for the OCT images of the fabrics was used for the analysis.

The results of the PCA transformation projected on the plane of the first two components (PC1–PC2) are shown in Figure 9 for a complete set Haralick features and $f_{P}$ feature, including also these not included in Table 2 and Table 3. Sample data are presented there as the cloud of points, the vectors beginning at the origin of the coordinate system are factor loadings which represent original feature variables. Most factor loadings of selected features are at a small angle to the PC1 axis, which confirms their high correlation and allows the representation of them using this component.

**Figure 10:**
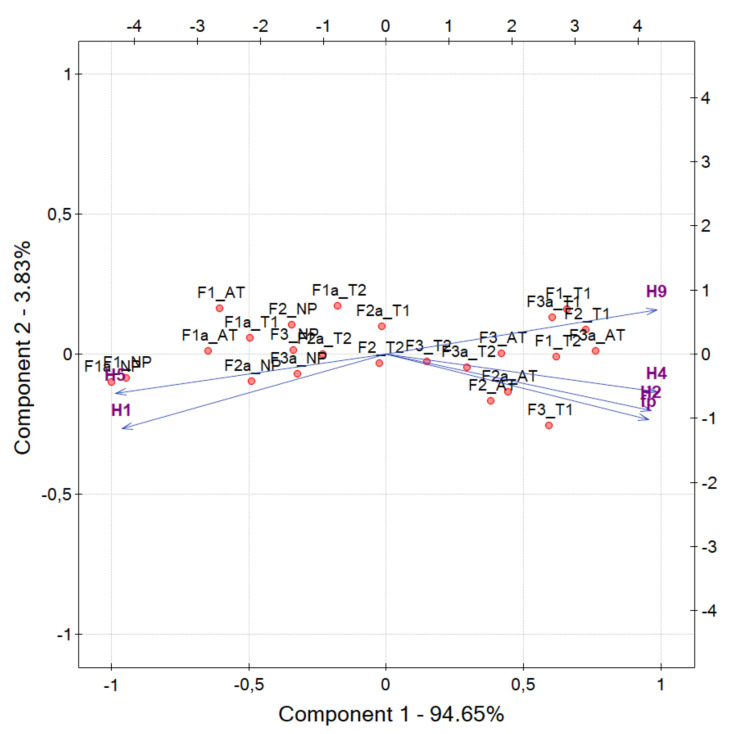
Score plot of strongly correlated textural features in $L_{P}$ layer of OCT images cast on the plane of principal components PC1, PC2 for different fabric abrasion tests. In addition to the original textile samples, fabrics subjected to laser ablation were also used for PCA analysis, marked in the plot with the suffix a. ($H_{1}$, $H_{2}$, $H_{4}$, $H_{5}$, $H_{9}$, $f_{P}$)—coordinates of the original feature space. F1, F2, F3—tested fabrics; NP—not pilled (control) sample; T1, T2—manual tests; AT—apparatus test.

**Figure 11:**
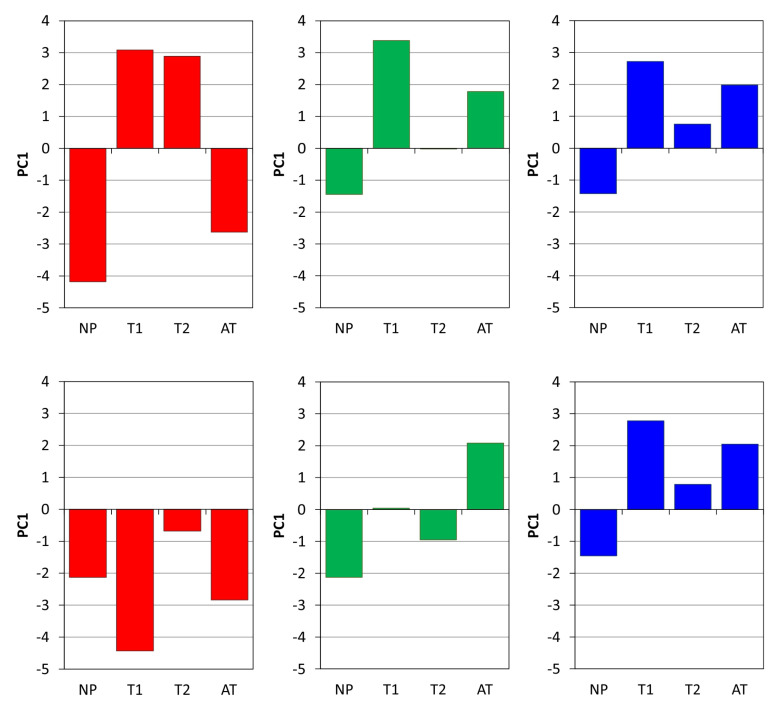
Plots of the PC1 component in the function of different pilling tests for various fabric types: F1, F2, F3—tested fabrics; $F1^{a}$, $F2^{a}$, $F3^{a}$—tested fabrics after the laser ablation; NP—not pilled (control) sample; T1, T2—manual tests; AT—apparatus test.

The value of PC1 component is a proposed pilling indicator. To improve the accuracy of representation by PC1 the correlated features $H_{1},H_{2},H_{4},H_{5},H_{9},f_{P}$ shown in Figure 8 PCA transform has been repeated only for them, with no participation of other Haralick features. The result of this operation is visualized in Figure 10. PC1 increases similarly to $H_{2}$, $H_{4}$, $H_{9}$ and $f_{P}$ when filling the layer $L_{P}$ with protruding fibers due to the partial detachment and pulling them out of the fabric layer after abrasion. The behavior of PC1 during tests carried out by the authors (T2, T1 and AT) is presented in Figure 11. The contribution of PC1 component in the textural feature vectors are reported in Table 4.

According to the description given in Section 2.2 of the article the manual test T1 is carried out using a higher pressure force and a rubbing surface with greater roughness than that used in T2, so the PC1 component after the test T1 achieves a higher value than in the case of the test T2 for the fabrics F1 and F2. The lowest PC1 value corresponds to the fabrics before test, which are not rubbed. The standard apparatus test AT gives lower PC1 values than T1. These results are confirmed by the observation of the relevant B-scans in Figure 7. The unexpectedly low values of PC1 for F1 fabric after the test AT are also confirmed by the lack of pills and protruding fibers inside of B-scan in the lowest row of Figure 7. All fiber formations in the $L_{P}$ layer formed during the test AT are finally detached, so this test is not reliable for pilling assessment.

The tested fabrics known as $F1^{a}$, $F2^{a}$ and $F3^{a}$ were subjected to laser ablation to change their surface properties and to reduce the pilling tendency. PC1 components of these ablated fabric types are shown in the bottom line of Figure 11. Laser modification can be seen to reduce PC1 compared to factory prepared surfaces, except for the fabric F3 with factory anti-pilling protection. PC1 values for fabric $F1^{a}$ become very low because F1 is an easily ablated fabric that completely loses its pilling tendency after laser treatment. The $F2^{a}$ fabric after surface laser ablation has mostly lower PC1 values than the corresponding values of F2 for the same types of pilling tests. However, only short-term tests, especially the T1 test, showed a change in the pilling tendency of these two fabrics. The PC1 values after apparatus test showed no significant changes in pilling compared to the state before laser modification, although experts have previously assessed that the quality of fabrics $F2^{a}$ and $F3^{a}$ has improved. The first component PC1 obtained after PCA transform of considered texture features in the fabric pilling layer can be used as a pilling measure for all types of tested fabrics.

## 4. Conclusions

This article has demonstrated, to the best of our knowledge, a new method for evaluating fabric pilling using the OCT imaging technique. Our approach allows improved observation and quantification of pilling in three dimensions, enabling pilling to be captured in the initial stage, even after a short period of low friction.

The most important feature of the proposed method is that the fuzzy mass of fiber is quantified objectively in three-dimensional OCT images, which will eliminate all errors related to subjective assessment by a man or uneven lighting of a fabric sample. The objective data can be obtained in the form of the proportion of protruding fibers or by using the texture parameters of the pilling layer. We have demonstrated that the texture variation accompanies different levels of pilling, and in addition, it can be expressed by means of one indicator. Thanks to the PCA transformation of selected Haralick features, together with fiber pixel fractions, a synthetic pilling index was obtained as the first principal component. This index can be used to objectively evaluate the degree of fabric pilling, although it requires comparative tests on a larger group of fabric samples as well as capture wide variation of pilling levels. Furthermore, the PC values could be similar, but not the same, for different groups of tests (various types of fabric, stitch construction, composition etc.). The proposed method may fail in the case of strongly reflecting material whose OCT image contains artifacts in the form of bright vertical streaks that prevent correct detection of pilling fibers. The algorithm of the method requires a much lower density of fibers (at least ten times) in the pilling layer than in the material layer so that the material layer can be detected correctly. The width of the pilling layer is determined arbitrarily by visual assessment of the upper limit of this layer in OCT images. Only protruding fibers forming structures wider than the OCT lateral resolutions 2dx and 2dy can be encountered as bright pilling voxels.

Current methods of fabric pilling assessment using expert knowledge or computer image analysis generally require intensive friction, using a pilling box, Martindale device, or the random tumble method to obtain pills on the tested fabric surface. The existing methods of pilling evaluation use mainly a flat material image, include no spatial information and require careful ambient lighting to obtain proper visibility of the pills by human or computer algorithms. It has been demonstrated that polyester fabrics may give a different picture of pilling trends as a result of different types of tests. It is due to the results of standardized tests do not always correlate well with end-use performance. These fabrics may lose pills under the influence of strong friction in the Martindale test and in this way the test result may be falsified. In this case, qualifying fabric as good quality is not allowed. In tests that detect and quantify pilling in the early stages, as proposed by us, it can be shown whether the fabric is more or less resistant to fiber detachment. Since these tests are short-term, they do not lead to mass detachment of fibers and pills. Therefore, these tests can be considered more reliable. Three-dimensional imaging of fabric surfaces using OCT offers a highly effective method for assessing pilling, due to the high resolution of the scans and the possibility of imaging individual fabric fibers, with the thickness above the device resolution limits. This technique may lead to a better understanding of the behavior of different textiles during normal use. As demonstrated, this OCT method is characterized by high sensitivity to the presence of fibers above the surface of the fabric, even when they do not appear after abrasion test, but naturally, as a result of habitual contact with other surfaces.

## Figures and Tables

**Table 1:** Characteristics of knitwear samples used in abrasion tests. The pilling grade refers to materials after testing with a Martindale device.

Name	Composition	Weight $\left[g/m^{2}\right]$	Pilling Grade	Laser Power [W]	Pilling Grade after Ablation
F1	100% polyester	240	5	14	5
F2	65% polyester	240	2	18	3
	35% polyacrylonitrile				
F3	68.1% cotton	240	2–3	16	3
	31.9% poliamid				

**Table 2:** Fraction $f_{P}$ of fiber voxels (bright pixels in OCT image) counted in the pilling layer $L_{P}$ of fabrics subjected to friction tests.

Textile	Not Pilled (Control) [%]	SD	Manual Test 1 [%]	SD	Manual Test 2 [%]	SD	Apparatus Test [%]	SD
F1	0.43	0.22	2.56	0.38	2.93	0.46	0.24	0.02
F2	0.90	0.06	2.54	0.30	1.88	0.34	2.67	0.14
F3	0.89	0.08	2.89	0.07	1.64	0.19	2.16	0.14

**Table 3:** Selected Haralick’s texture features extracted using GLCM of layer $L_{P}$ in the OCT image.

Feature	Textile	Not Pilled (Control)	SD	Manual Test 1	SD	Manual Test 2	SD	Apparatus Test	SD
**Energy**	F1	0.0045	0.0006	0.0021	0.0002	0.0024	0.0002	0.0036	0.0006
$H_{1}$	F2	0.0034	0.0003	0.0021	0.0004	0.0032	0.0004	0.0030	0.0004
	F3	0.0035	0.0005	0.0027	0.0006	0.0030	0.0007	0.0025	0.0003
**Contrast**	F1	17.08	0.46	64.39	7.56	69.24	7.05	24.60	1.19
$H_{2}$	F2	33.08	3.26	75.53	16.37	47.79	4.97	68.24	3.78
	F3	41.83	1.24	87.57	1.97	62.24	8.24	68.77	2.89
**Variance**	F1	33.43	4.28	177.85	13.77	181.26	20.83	55.89	14.90
$H_{4}$	F2	84.43	11.99	196.26	35.95	121.02	17.00	175.34	12.64
	F3	94.49	8.26	205.17	5.96	151.98	28.13	174.91	17.20
**Homogeneity**	F1	0.299	0.006	0.241	0.005	0.249	0.001	0.283	0.002
$H_{5}$	F2	0.276	0.005	0.244	0.008	0.270	0.007	0.257	0.005
	F3	0.280	0.002	0.259	0.006	0.266	0.007	0.258	0.003
**Sum average**	F1	122.05	7.01	115.82	11.46	109.41	15.92	65.58	4.67
$H_{6}$	F2	123.38	6.34	118.30	10.90	125.57	6.52	100.63	8.37
	F3	84.50	1.29	65.98	4.99	90.78	5.11	72.59	3.18
**Entropy**	F1	8.35	0.18	9.86	0.10	9.71	0.15	8.76	0.25
$H_{9}$	F2	8.96	0.16	9.89	0.30	9.15	0.18	9.37	0.18
	F3	8.93	0.18	9.51	0.23	9.31	0.33	9.54	0.15

**Table 4:** Contributions of the PC1 principal component in the representation of observations in the feature space for: $\left(H_{1},H_{2},H_{4},H_{5},H_{9},f_{P}\right)$. F1, F2, F3—tested fabrics; $F1^{a}$, $F2^{a}$, $F3^{a}$—tested fabrics after the laser ablation.

Textile	Not Pilled [%]	Manual Test 1 [%]	Manual Test 2 [%]	Apparatus Test [%]
F1	99.43	95.85	98.17	96.36
F2	95.37	99.23	25.52	89.70
F3	98.44	91.11	85.43	99.03
$F1^{a}$	99.49	99.15	71.26	99.90
$F2^{a}$	97.89	14.18	99.48	94.56
$F3^{a}$	95.93	96.41	94.70	99.36

## Abbreviations

The following abbreviations are used in this manuscript:

OCT	optical coherence tomography
SS-OCT	swept-source OCT
MEMS	Micro-Electro-Mechanical Systems
SD-OCT	spectral domain OCT
GLCM	Gray-Level Co-occurrence Matrix
PN-EN	Polish Norm introducing the European Norm
PN-EN ISO	Polish Norm introducing the International Norm
ISO	International Organization for Standardization
SPI	Southampton Photonics Inc.
ASTM	American Society for Testing and Materials
PC	personal computer
PCA	principal component analysis
PC	principal component
PC1	first principal component
PC2	second principal component
*NP*	not pilled
*AT*	apparatus test
*T*1	Test 1
*T*2	Test 2
